# Supplementary material for: Metal-organic frameworks with photocatalytic bactericidal activity for integrated air cleaning
Source: Nat Commun. 2019 May 16;10:2177. doi: 10.1038/s41467-019-10218-9 (PMC6522529; doi:10.1038/s41467-019-10218-9)
Supplement: Supplementary file 1 — Supplementary Information [file 41467_2019_10218_MOESM1_ESM.pdf]

Supplementary Information

Metal-organic frameworks with photocatalytic bactericidal activity for integrated air cleaning

Li et al.

Supplementary Methods

Anatase TiO_2 (25 nm, 99.8%) and ZnO (50 nm, 99.8%) were purchased from Macklin Biochemical Co. Ltd. Nutrient broth (NB, BR) and nutrient agar (NA, BR) were received from Beijing Aoboxing biotech Company (Beijing, China). *E. coli* (CPCC 100522 (Chinese Academy of Medical Sciences) were selected as bacterial strains. All other chemicals and solvents were purchased from commercial suppliers including J&K Scientific Co. Ltd., Sigma-Aldrich Co. Ltd., Energy Chemical Co. Ltd., Beijing Chemical Reagent Company, and used without further purification. Non-woven fabric employed in this work is the spunlaced non-woven fabric with polyethylene (PE) and polyethylene terephthalate (PET) (1:2). It has a fiber diameter of ca. 12 μm, thickness of 0.190 mm, density of 1.4 g cm^{-3}, maximum pore size of 116.6 μm, average pore size of 56.1 μm and porosity of 69.7%.

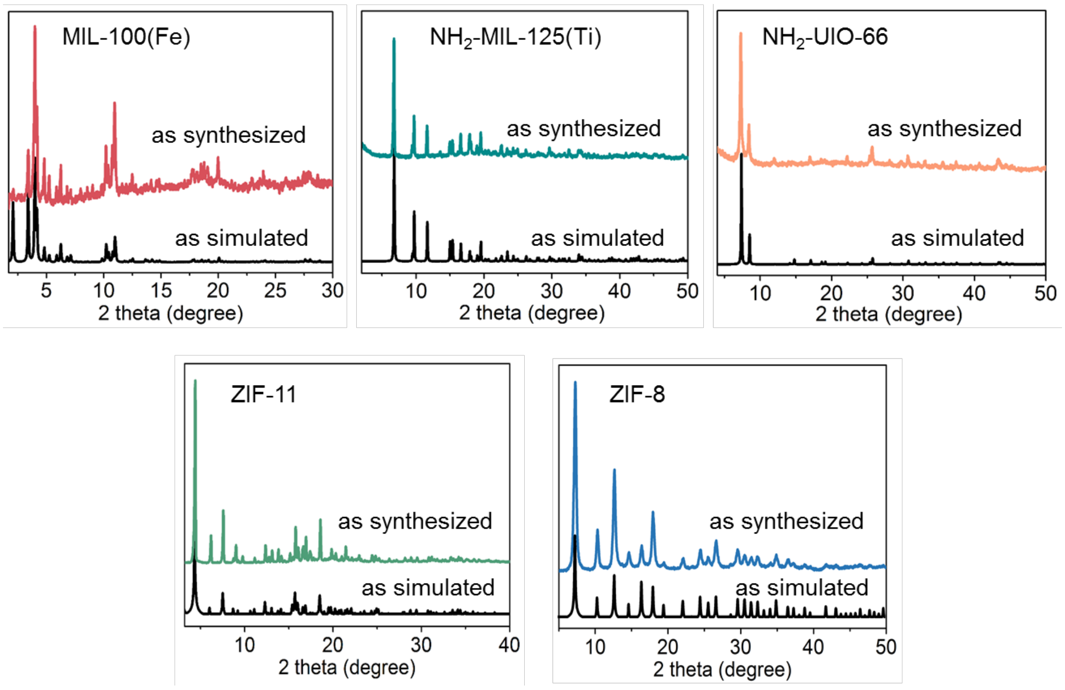

Supplementary Figure 1 PXRD patterns of MIL-100(Fe), NH_2-MIL-125(Ti), NH_2-UIO-66, ZIF-11 and ZIF-8.

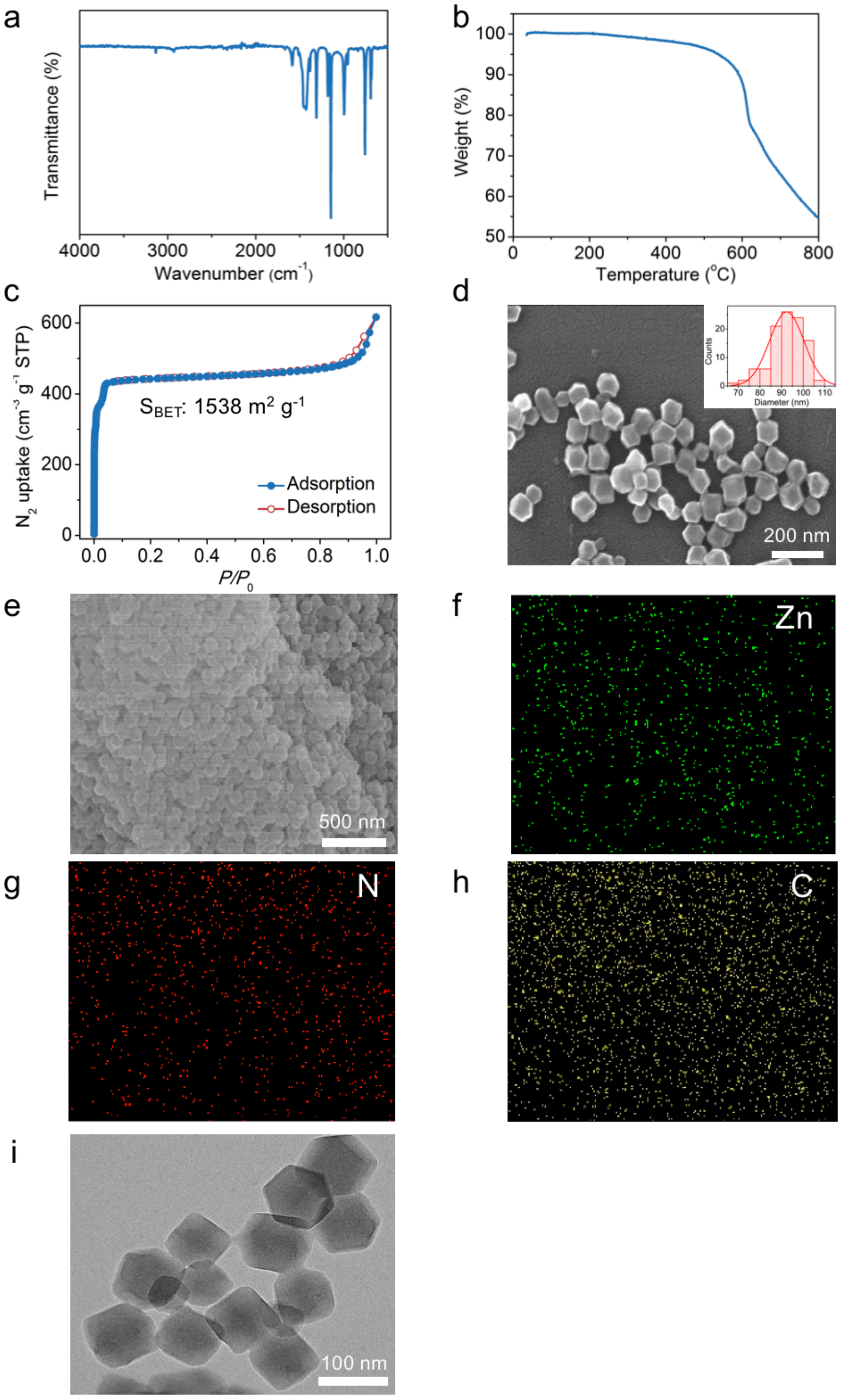

Supplementary Figure 2 **a** FT-IR spectra, **b** TG curve, **c** N_2 sorption isotherms measured at 77 K, **d** SEM image and size distribution (insert), **e-h** elemental mapping analysis, **i** TEM image of ZIF-8.

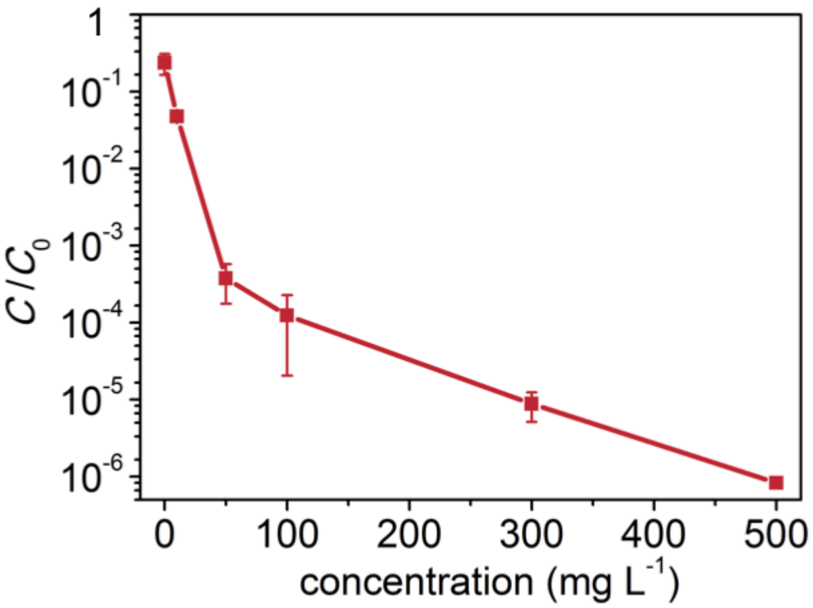

Supplementary Figure 3. Photocatalytic disinfection performance of ZIF-8 at different dosage levels. The error bars are calculated *via* repeating the measurements for three times.

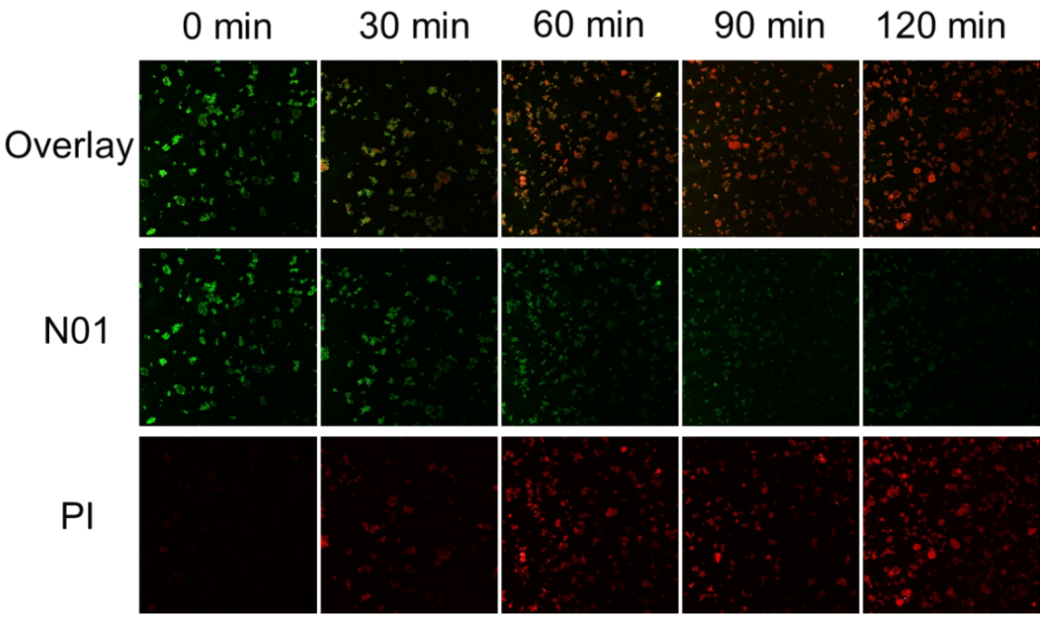

Supplementary Figure 4. Confocal fluorescence images of *E. coli* stained with N01 (live cells, green fluorescence) and PI (dead cells, red fluorescence), after cells (10^7 CFU mL^{-1}) photocatalytically treated by ZIF-8 (500 mg L^{-1}) under sunlight irradiation for 0 min, 30 min, 60 min, 90 min and 120 min respectively.

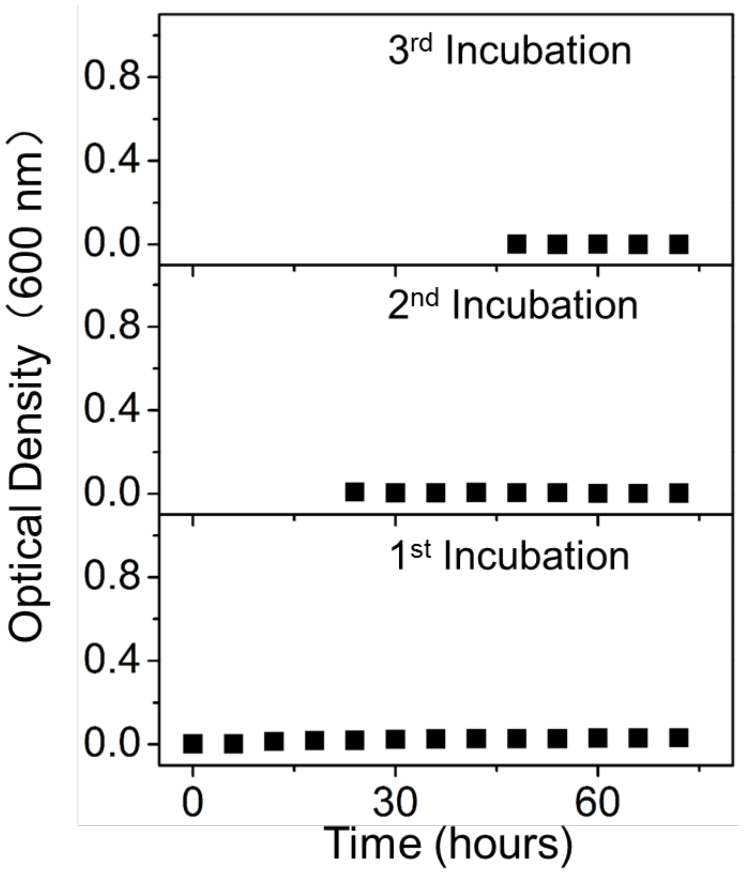

Supplementary Figure 5. Regrowth test of *E. coli* after photocatalytically treated by ZIF-8. The 1st, 2nd and 3rd incubation indicate regrowth at 0 h, 24 h and 48 h after photo disinfection experiment.

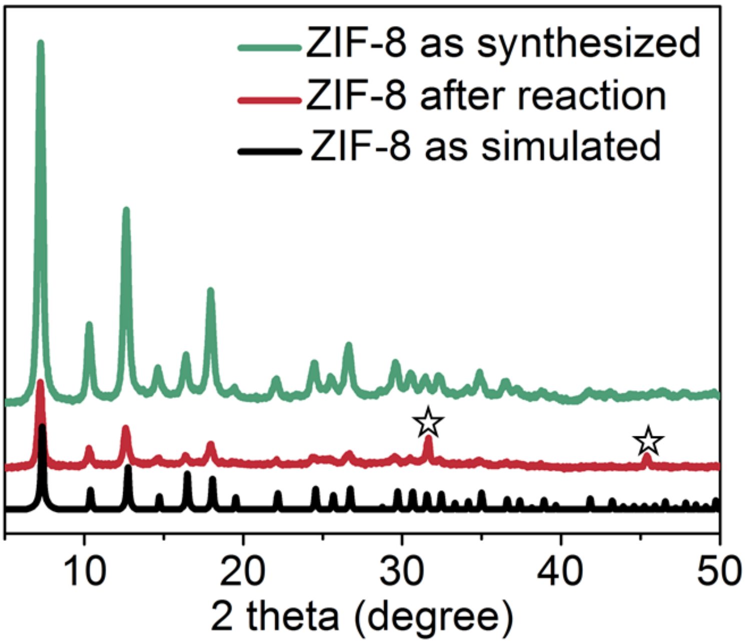

Supplementary Figure 6. PXPD patterns of simulated and as-synthesized ZIF-8 along with ZIF-8 after photocatalytic reaction. The peaks marked with pentagram represent the characteristic diffraction signals of NaCl.

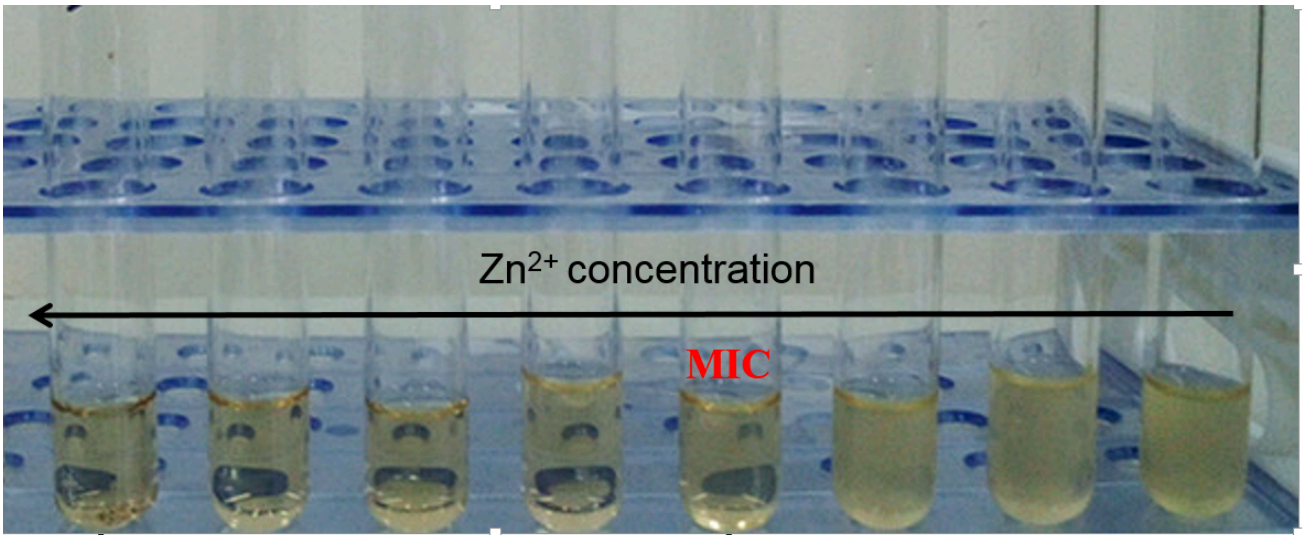

Supplementary Figure 7. The optical images showing MIC measurement results.

Supplementary Table 1 The values of MIC and MBC of Zn^{2+} against *E.coli* strain.

	MIC (mg/L)	MBC (mg/L)
	E.coli	
Zn^{2+}	31.25	250

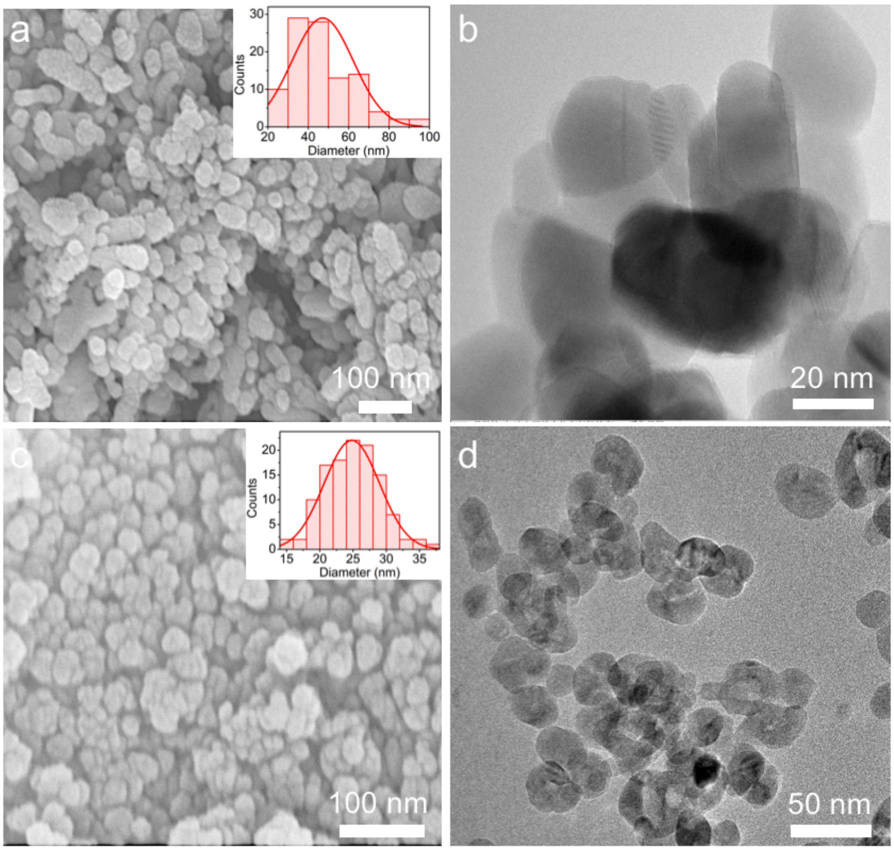

Supplementary Figure 8. a SEM and **b** TEM images of ZnO, **c** SEM and **d** TEM images of TiO_2.

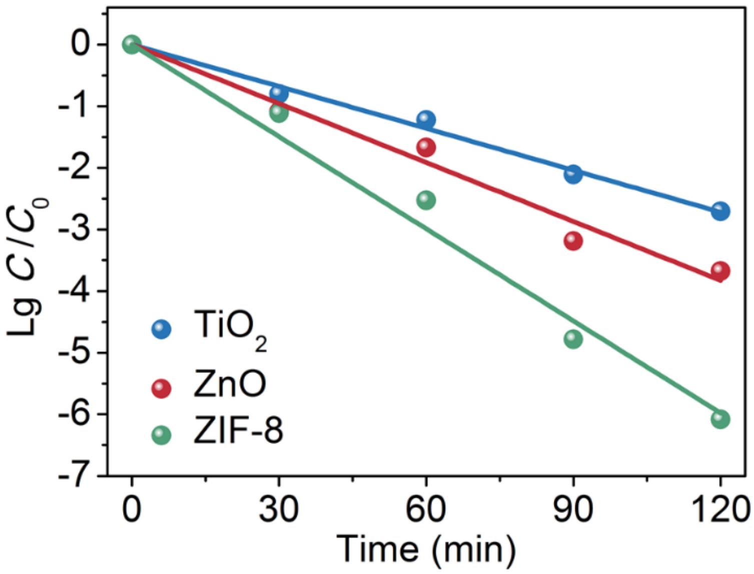

Supplementary Figure 9. Inactivation kinetics on TiO_2, ZnO and ZIF-8

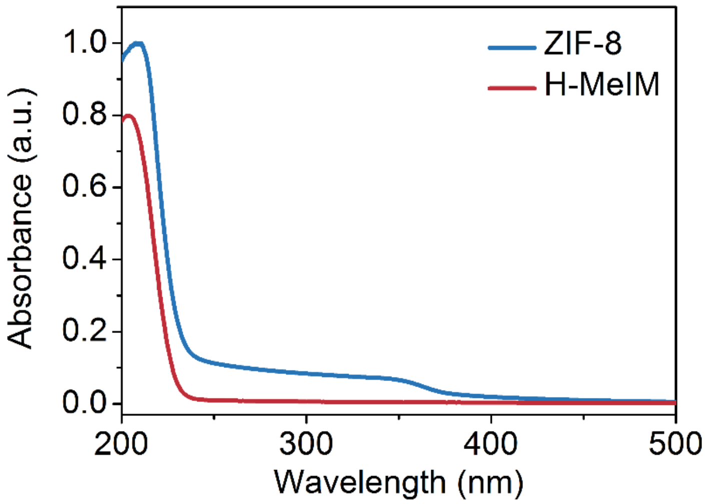

Supplementary Figure 10. UV−vis diffuse reflectance spectra of ZIF-8 and H-MeIm.

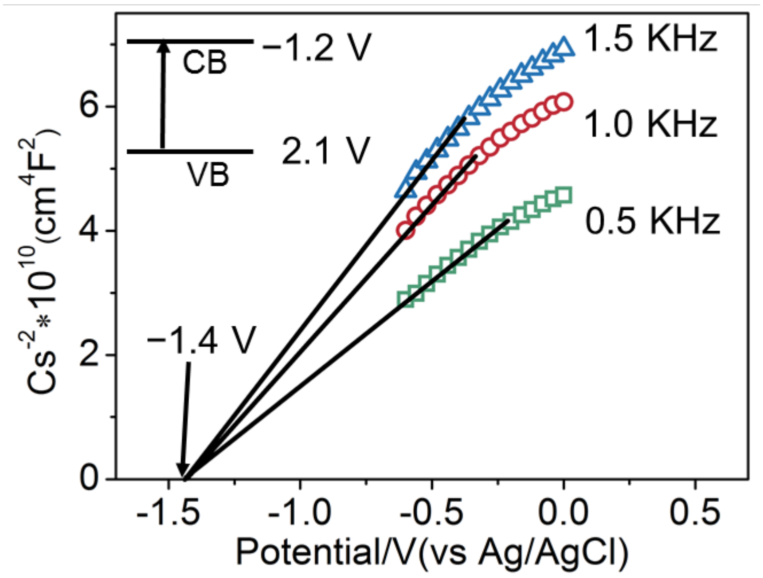

Supplementary Figure 11. The Mott–Schottky plots of ZIF-8.

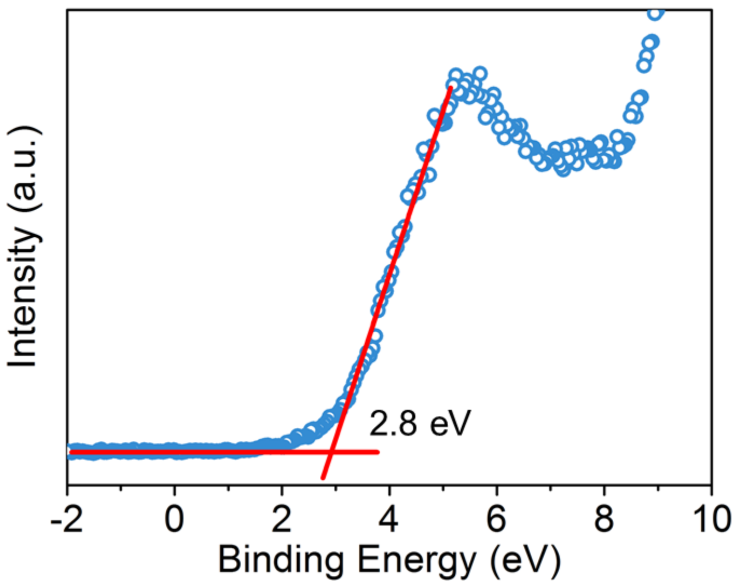

Supplementary Figure 12. The VB-XPS spectra of ZIF-8.

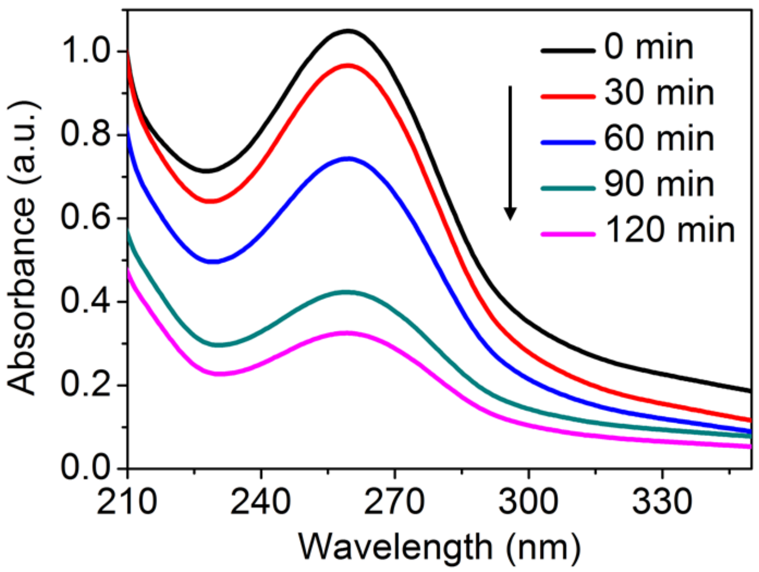

Supplementary Figure 13. Time-dependent absorption spectra of nitroblue tetrazolium (NBT) for •O_2^- detection

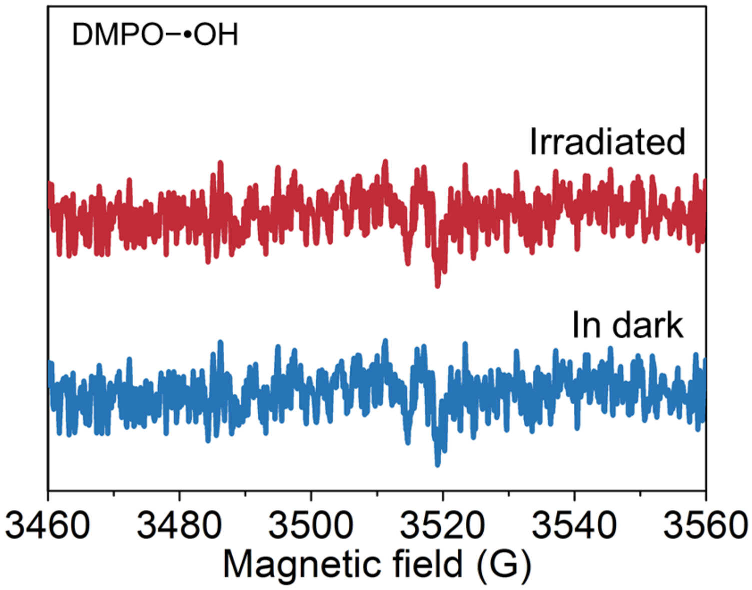

Supplementary Figure 14. EPR spectra of DMPO−•OH recorded in ZIF-8 aqueous solution suspension under light and dark conditions.

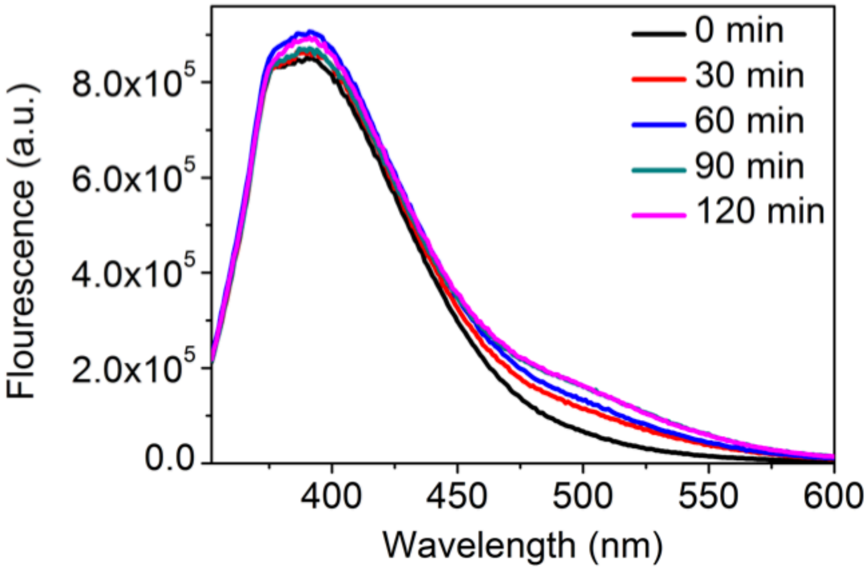

Supplementary Figure 15. Time-dependent fluorescence spectra of generated 7-hydroxycoumarin in ZIF-8 reaction system for •OH detection. 7-hydroxycoumarin could emit fluorescence at 455 nm when excited at 332 nm.

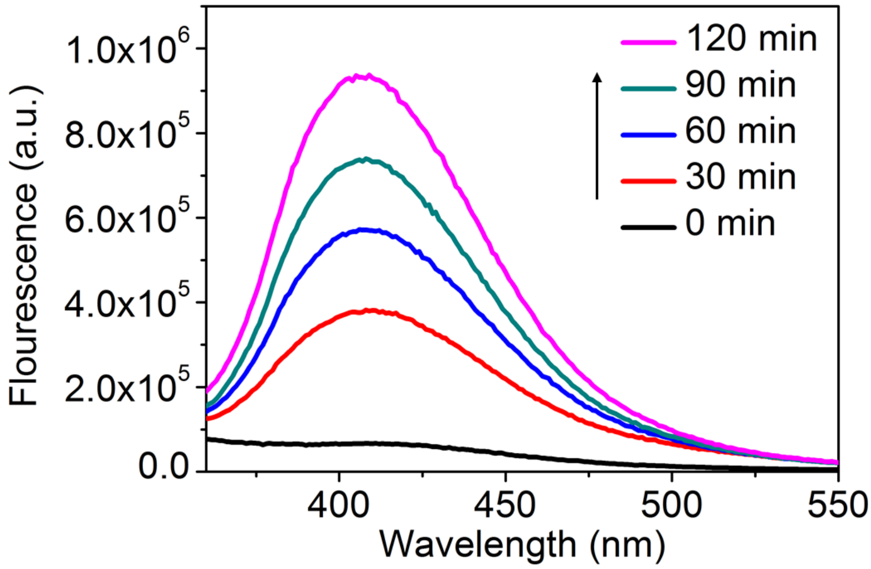

Supplementary Figure 16. Time-dependent fluorescence spectra of produced p-hydroxyphenylacetic acid dimer in reaction system for H_2O_2 detection.

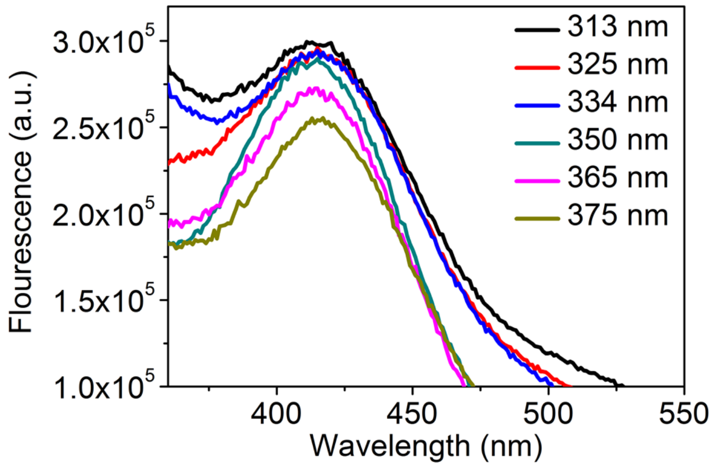

Supplementary Figure 17. Fluorescence spectra of produced p-hydroxyphenylacetic acid dimer in ZIF-8 reaction system. ZIF-8 suspensions were irradiated by single-wavelength incident light with wavelength ranging from 313 to 375 nm respectively.

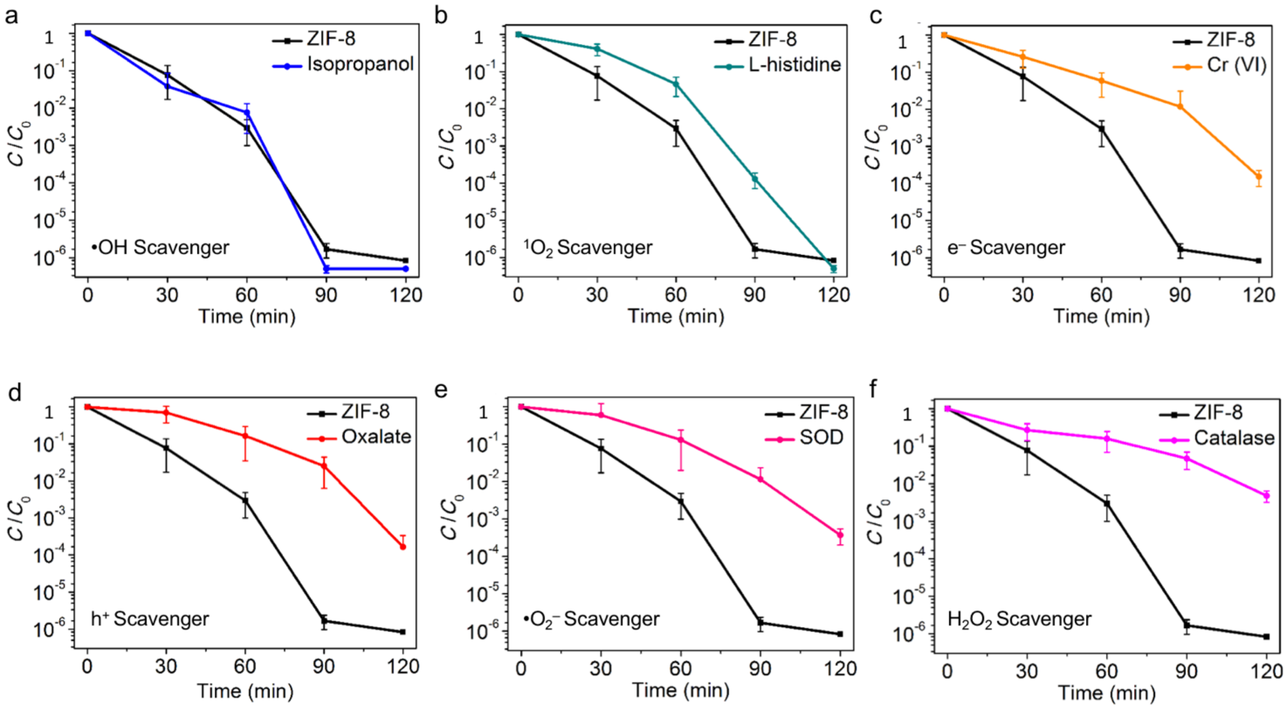

Supplementary Figure 18. a Photocatalytic disinfection performance with scavenger isopropanol to quench photo generated •OH, **b** L-histidine to quench photo generated $^1O_2$, **c** sodium chromate to quench photo generated e^-, **d** sodium oxalate to quench photo generated h^+, **e** SOD to quench photo generated $•O_2^-$, **f** catalase to quench photo generated H_2O_2. The error bars are calculated *via* repeating the measurements for three times.

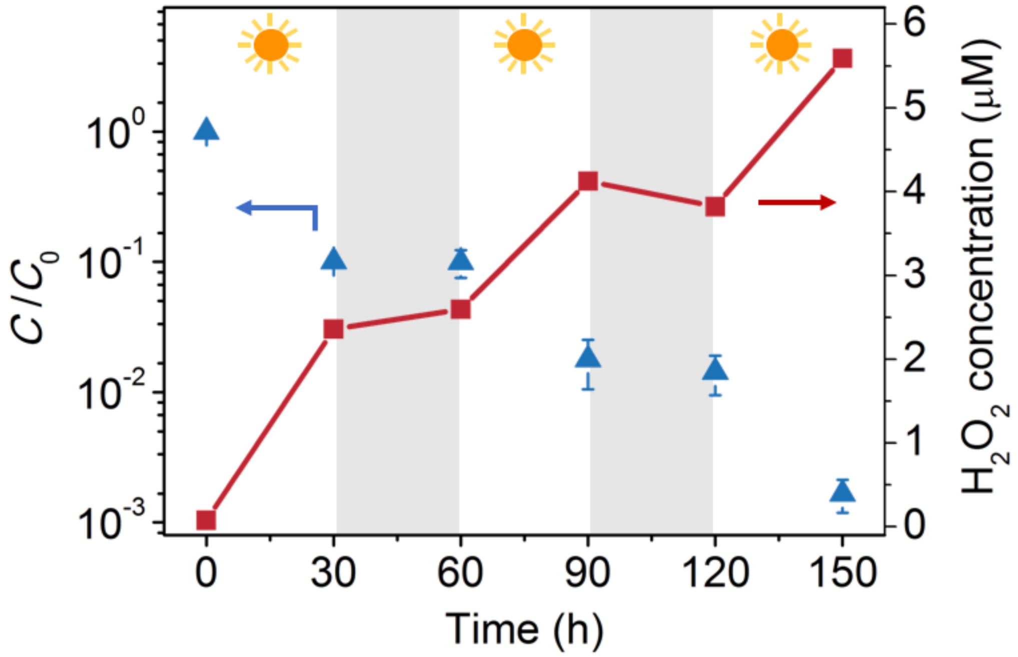

Supplementary Figure 19. Disinfection performance of ZIF-8 and the concentration of generated H_2O_2 versus time under the alternated light and dark conditions (irradiation in white and dark periods in gray). The error bars are calculated *via* repeating the measurements for three times.

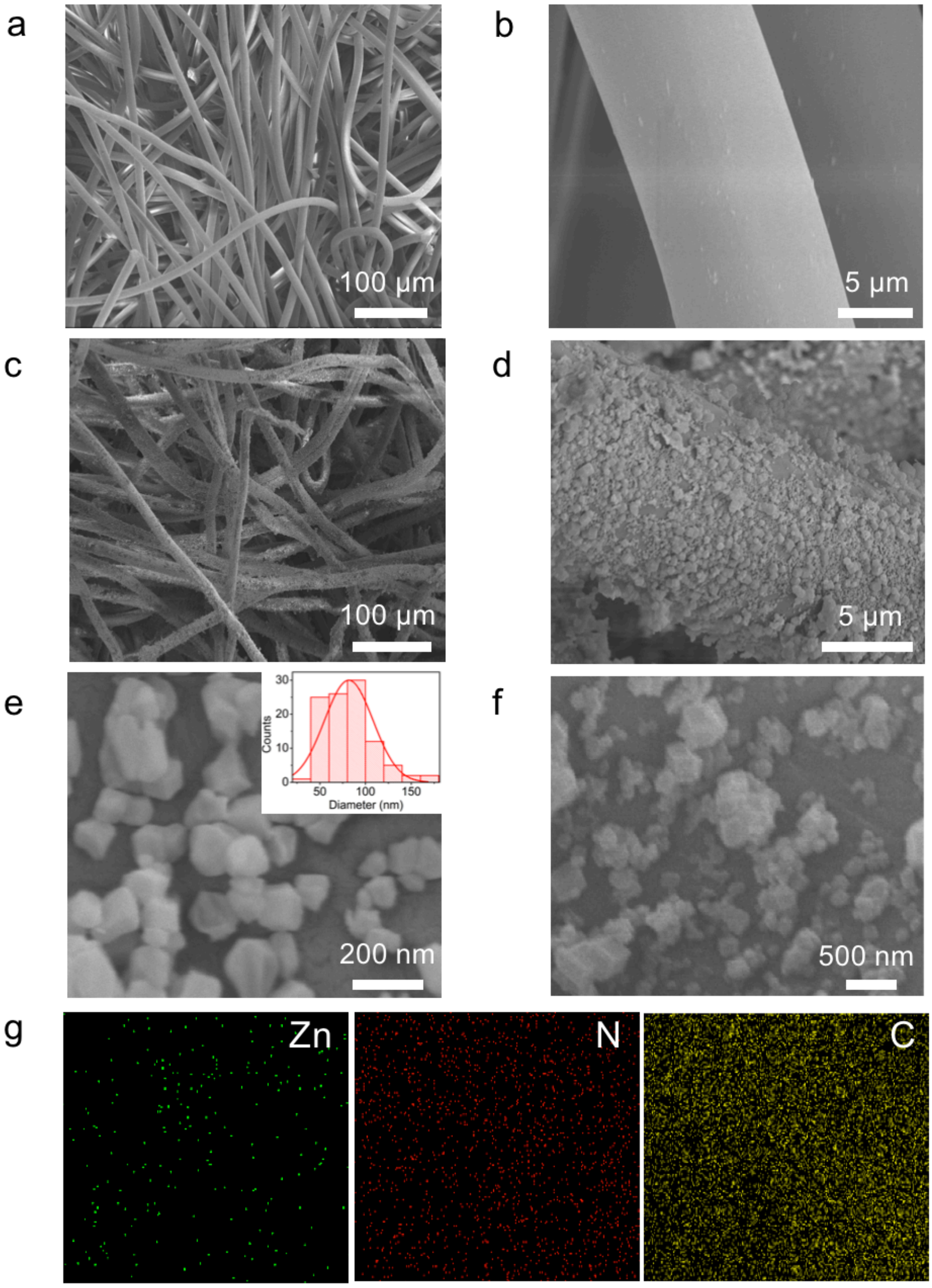

Supplementary Figure 20. a, b SEM images of NWF, **c, d, e** SEM images of MOFilter and size distribution of ZIF-8 particles grown on fibers (insert), **f, g** corresponding elemental mapping images.

Supplementary Table 2. The weight change of MOFilter after continuously bended and rubbed for 1000 times.

Sample	m_1 (mg)	m_2 (mg)	Δm (mg)
1	223.97	223.65	0.32
2	213.17	213.02	0.15
3	207.57	207.41	0.16
4	218.76	218.51	0.25
5	214.88	214.27	0.61
6	212.49	212.33	0.16
7	219.85	219.80	0.05
8	211.33	211.09	0.24
9	210.22	310.13	0.09
10	233.55	233.19	0.36

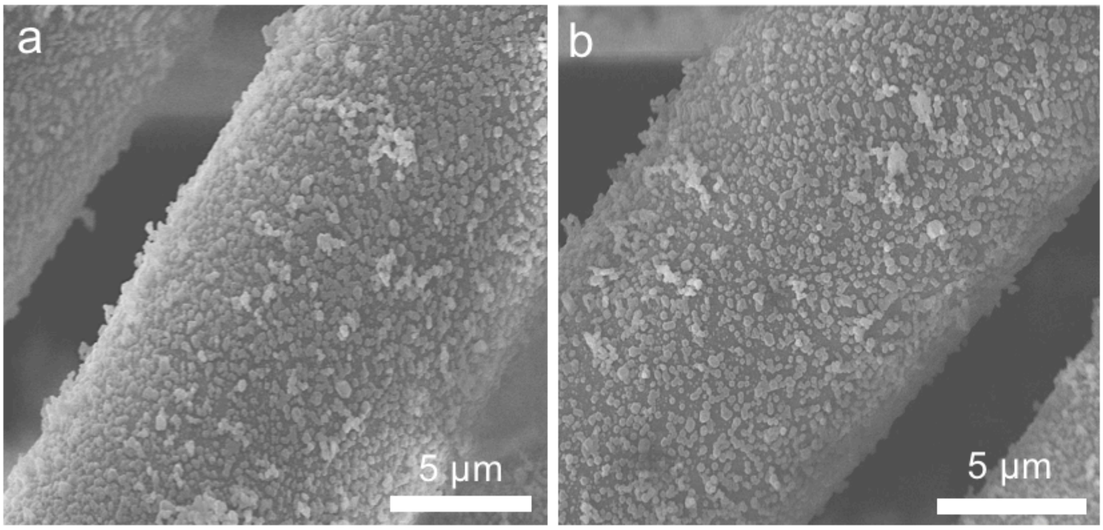

Supplementary Figure 21. SEM images of MOFilter **a** before and **b** after bended and rubbed repeatedly for 1000 times.

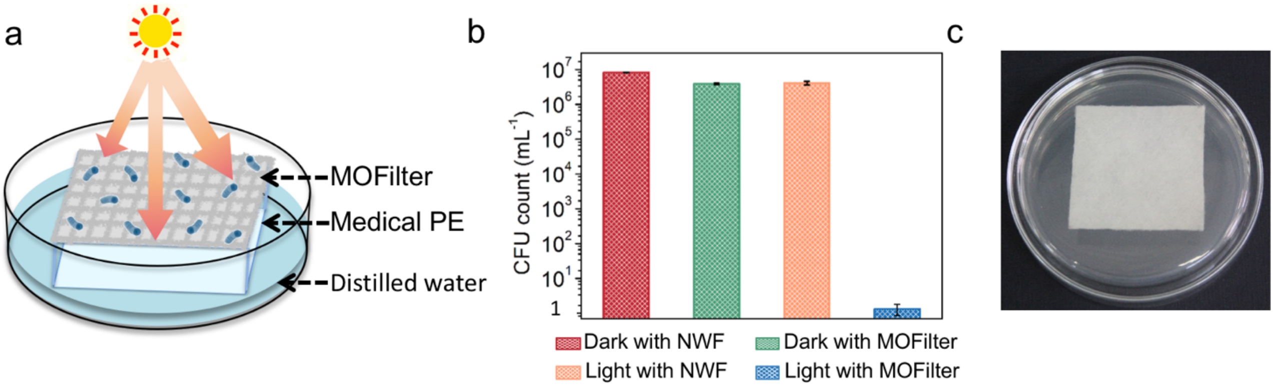

Supplementary Figure 22. a Schematic representation of the test method for surface disinfection under light irradiation. **b** Comparison of the number of viable cells present in eluent used for collecting living bacteria on MOFfilter after reaction. **c** The bacterial colonies residual on eluent-treated MOFilter after photocatalytic reaction. The error bars are calculated *via* repeating the measurements for three times.

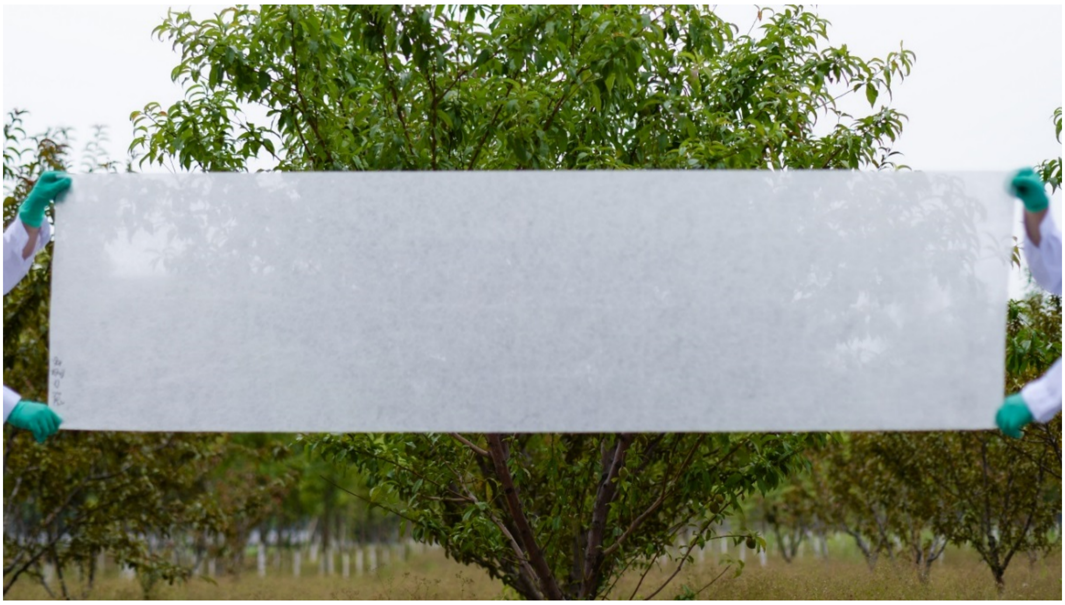

Supplementary Figure 23. The optical image of MOFilter with a large area.

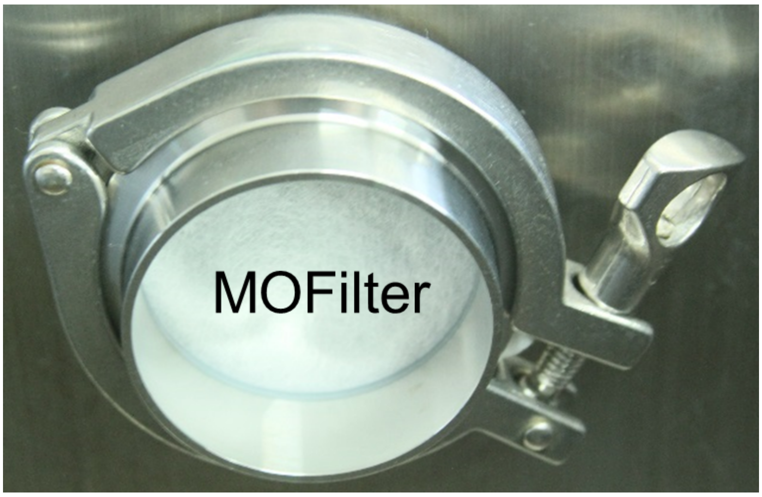

Supplementary Figure 24. The optical image of MOFilter fixed in device.

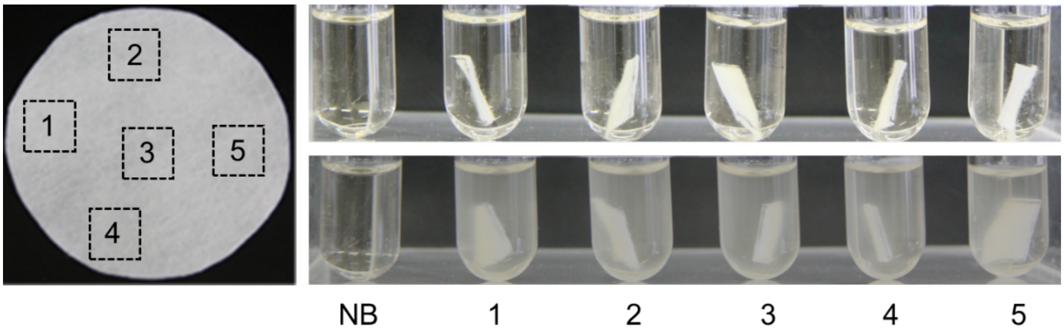

Supplementary Figure 25. After photocatalytic reaction, MOFilter and NWF (samples represented by *light with MOFilter* and *light with NWF* respectively in Figure 4e) were cut into five pieces respectively, and immersed in nutrient broth (NB) solution for 20 h at 37 ^{o}C for confirmation of residual viable cells on filter (top row for MOFilter and bottom row for NWF).

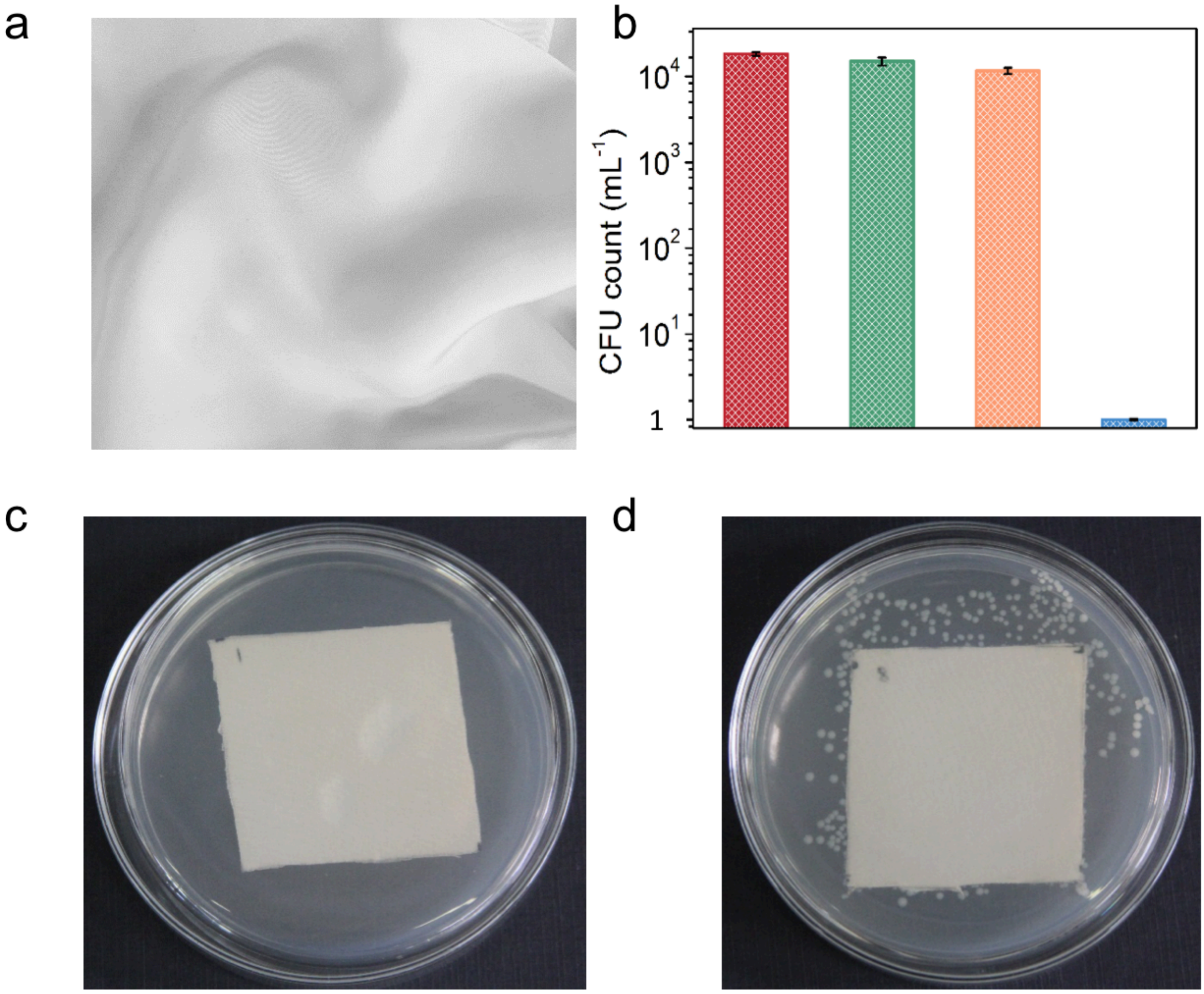

Supplementary Figure 26. **a** Optical image of silk substrate used for MOFilter fabrication. **b** Comparison of the number of viable cells present in eluent used for collecting living bacteria on MOFilter (silk as substrate) after reaction. **c, d** The bacterial colonies residual on eluent-treated MOFilter (silk as substrate) **c** and silk substrate **d** after photocatalytic reaction. The error bars are calculated *via* repeating the measurements for three times.
